# Wilson maxillary curve analyzed by cbct. a study on normocclusion and malocclusion individuals

**DOI:** 10.4317/medoral.18291

**Published:** 2013-03-25

**Authors:** José M. Barrera, José M. Llamas, Eduardo Espinar, Carlos Sáenz-Ramírez, Vanesa Paredes, Juan C. Pérez-Varela

**Affiliations:** 1 Associate Professor of Orthodontics. University of Seville; 2 Professor in Master of Oral Surgery. University of Seville; 3 Assistant Professor of Orthodontics. University of Valencia; 4 Associate Professor of Orthodontics. University of Santiago de Compostela

## Abstract

The anatomy of dental compensation curve in the frontal plane described by George H. Wilson is one of the occlusal determinants of orthodontic treatment. However, there is few published comparing malocclusion and normocclusion individuals. Objectives: The aim of this study is to compare the curve of Wilson at first and second maxillary molars, normocclusion pattern and malocclusion pattern, with and without bilateral posterior crossbite, using angular references in CBCT studies. Material and Methods: We analyzed 10 cases of malocclusion with bilateral posterior crossbite, 10 cases of malocclusion without bilateral posterior crossbite and 10 cases with non orthodontic normocclusion (patients who underwent cone beam study for other reasons than orthodontic). All of them were adults, more than 19 years. Angular variables from left and right axis (line connecting the occlusal and furcation groove) of first and second molars towards a perpendicular to the frontal palate were measured. There was carried out an Anova test, Bonferroni analysis and Levene´s statistics. Results: The descriptive analysis of the results shows an average values of total maxillary curve of Wilson for first molars (sum of left and right angle) of 8.1° for normocclusion group, 0.4° for the malocclusion pattern with bilateral posterior crossbite and 16.9° for the malocclusion pattern without this alteration. The mean differences was statistical significant (P<0,042) between between malocclusion pattern groups with and without crossbite . Conclusion: The curve of Wilson, measured at maxillary first molars in patients with bilateral posterior crossbite is more concave than the other groups, suggesting no dentoalveolar compensations.

** Key words:**Wilson curve, CBCT, buccal posterior occlusion, posterior crossbite.

## Introduction

Occlusion in orthodontics has been studied looking for a benefit to the population, therefore taking an importan place in finishing requirements (1-5). Many theories support (6,7) different occlusion schemes, often with contradictory concepts, but there are few studies based on scientific evidence, trying to clarify and obtain clinical applicability. This is specially remarked regarding the frontal view at bucolingual posterior cusps, as we know that the occlusal surfaces of the molars do not follow a single plane. In 1911, George H. Wilson described this phenomenon with a curve, described as a compensatory curve to avoid possible balancing interferences. This curve must be concave in the mandible arch concave and convex in the maxillary arch. Therefore palatal and buccal cusps of posterior teeth contact in a functional way.

Analyzing a sample of 120 normocclusion cases, Andrews (8) described six keys of occlusion used to develop the Straight Wire Appliance as we basically use it actually, conforming a practical finishing occlusal guide. The fourth key is related to the curve of Wilson, describing the posterior inclination of the crowns of the posterior upper teeth as a concave curve, setting the molars with a lingual torque.

The American Academy of Orthodontists in the ABO Grading Models System established maximum intercuspation without balancing interferences, with a plane between upper molar cusps and a mandibular curve slightly concave with the lingual cusp descending 1 or 2 mm compared to the buccal ones.

Wilson`s curve has been the subject of studies related to the change in angulation during growth (9), as an indicator of post-expansion (10) maxillary stability and even as a etiological factor of temporomandibulars disorders (11), but the emergence of the new CBCT records allows us to perform a more exhaustive study of the curve to try to quantify it in different normocclusion and malocclusion patterns. Alqerban et al. (12) reported the applicability of the Cone-beam computed tomography in the position, inclination of the teeth, and it relation to adjacent structures. It makes CBCT a very effective tool in numerous studies and research. There is a general consense regarding the different buccolingual inclination in the posterior teeth when a upper transversal compression exists, defending a dentoalveolar compensation that brings the upper posterior teeth to a more positive torque, therefore creating a convex Wilson curve in the upper denture. Therefore, the aim of this research is to verify that knowledge using CBCT images, comparing malocclusive adult cases with and without maxilar bilateral posterior crossbite and normocclusives non orthodontic adult individuals.

## Material and Methods

The sample consisted of 30 adult individuals, aged between 19 and 55 years. They were divided into three groups: 10 cases of malocclusion with bilateral posterior firs molars crossbite, 10 cases of malocclusion without crossbite, and 10 normocclusives non orthodontic individuals. The exclusion criteria of malocclusion groups were the absence of maxillary and mandibular first or second molars, the presence of unilateral posterior crossbite or premolars agenesis. Exclusion criteria in the normocclusive group were described by Wadhwa L, Utreja A, Tewari (13) age below 18 years, absence of a bilateral molar or canine Angle class I, positive or negative arch discrepancy exceeding 2 mm, anterior or posterior crossbite, overjet more than 2mm or less than 0 mm, overbite greater than 4 mm or less than 2 mm, more than 15º rotations in posterior and anterior teeth, without previous orthodontic treatment.

**Figure 1:**
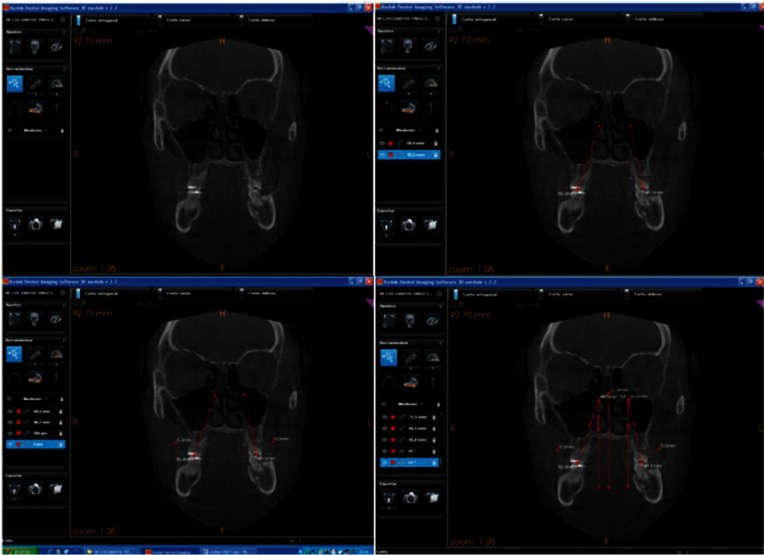
Angular measurements on Cone Beam method.

**Figure 2:**
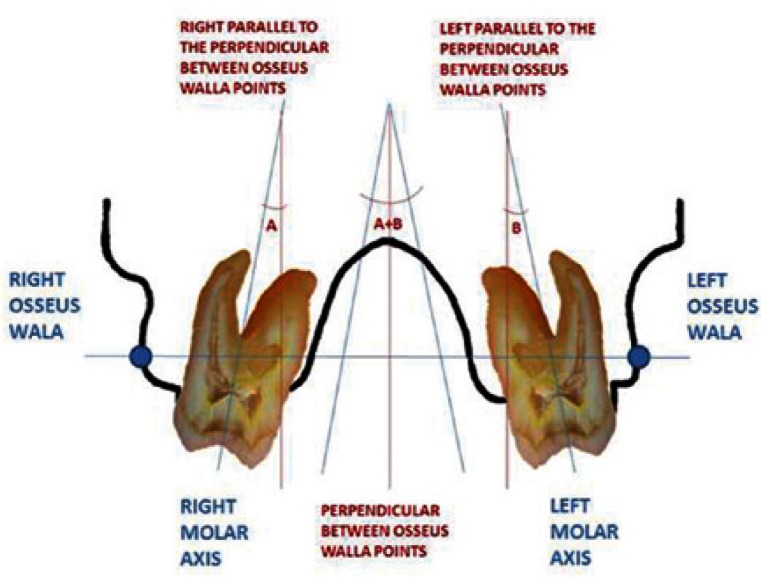
Outline of the procedure.

The records were taken in a Kodak 9500 3D machine and analyzed with the Kodak ® KDSI 3D software module. We made frontal sections cuts in maximum contacts of first and second molars. For angular measurement of the curve of Wilson we traced molar axis (line connecting the occlusal groove and furcation) of first and second left and right molars. We used a line trough right and left bone WALA point (modifying original WALA point by Andrews defined as the highest prominence of the soft tissue of the buccal alveolar crest), creating a reference line. A perpendicular to that reference line was also traced. It allows us to measure the inclination of left and right molar and finally as can be seen in figures 1 and 2.

It was performed a descriptive analysis of data and the following inferential analysis to compare the mean differences : analysis of variance (ANOVA), post-hoc test (Bonferroni analysis) and Levene´s statistics .

## Results

The descriptive analysis of the results shows an average values of total maxillary curve of Wilson for first molars (sum of left and right angle) of 8.1° for normocclusion group, 0.4° for the malocclusion pattern with bilateral posterior crossbite and 16.9° for the malocclusion pattern without this alteration. The values for total maxillary curve of Wilson second molars was 24.9° for normocclusion group, 32.7° for the malocclusion pattern with bilateral posterior crossbite and 25.4° for the malocclusion pattern without this alteration ( Table 1).

**Table 1:**
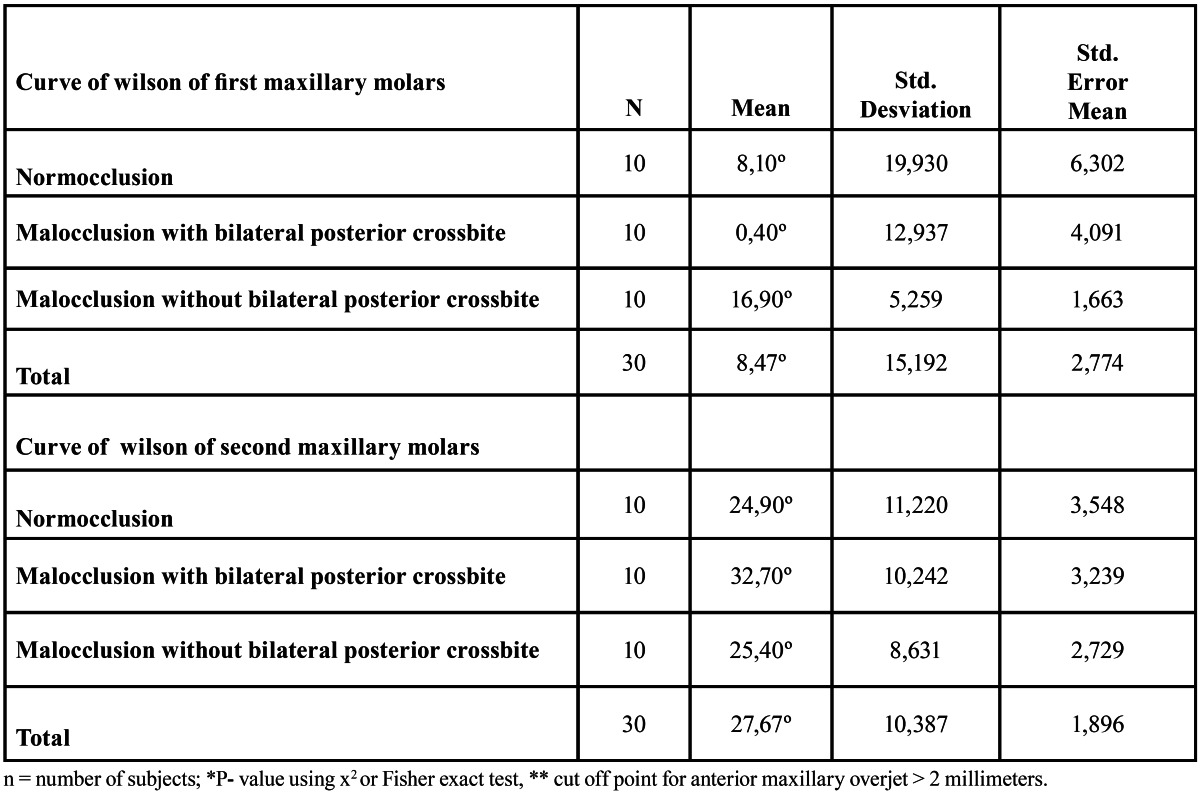
Descriptive analysis.

**Figure 3:**
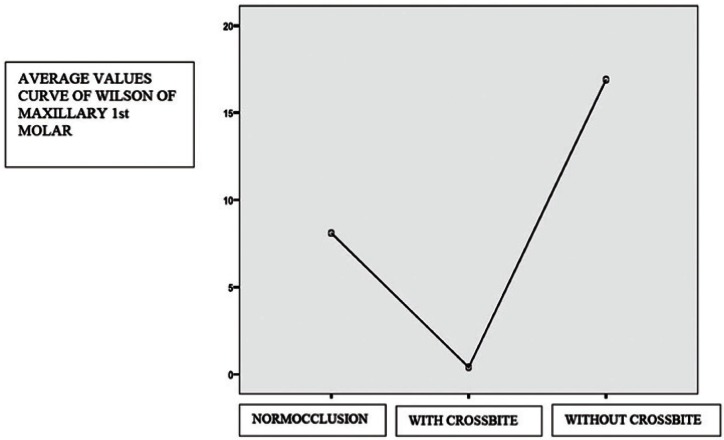
Mean of the difference between the groups.

Regarding inferential analysis (ANOVA, Bonferroni post-hoc tests and Levene´s statistics) are only statistically significant differences in the curve of wilson of first molars between the group with and without bilateral posterior crossbite 0.046 p <0.05 (Fig. 3). The statistically significant mean difference can be seen in  Table 2.

**Table 2:**
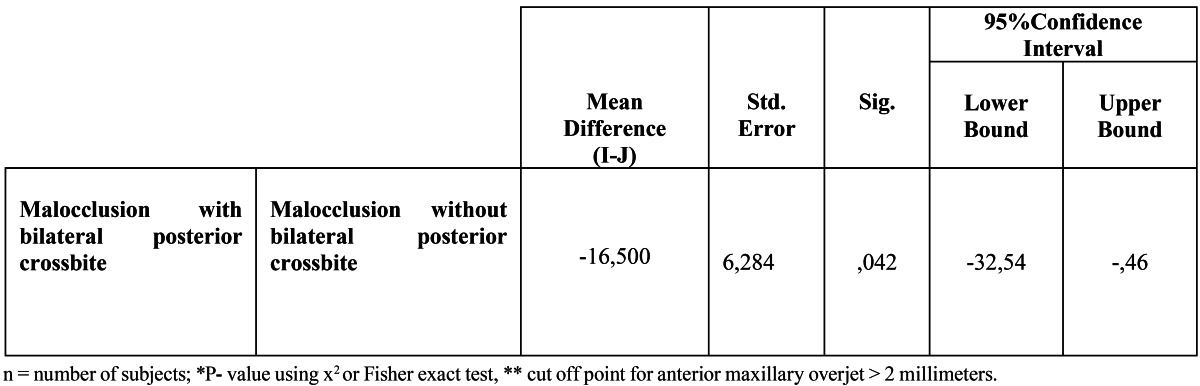
Post-hoc bonferroni´s test.

## Discussion

The main finding of this study showed a concave first molars curve of Wilson in malocclusive patients with bilateral posterior crossbite while a slightly convex curve was seen in the other two groups. The curve of second maxillary molars, however, shows convexity in the three groups, but being more pronounced in patients with bilateral posterior firs molars crossbite. We found, therefore, different results that published by Handelman et al (10), concerning molar inclinations, despite they found no statistically differences between the experimental and the control group before treatment.

The angulation of the first and second molars in our normocclusive sample (8.1 ° and 24.9 °) is similar to other studies with normocclusive patients (14-18), concerning the a buccal inclination of the maxillary molars. According to the study by Marshall et al. (9) maxillary first molars upright with age palatally (from 7.5 to 26.4 years). Our study is made in non growing patients, therefore these findings can not been assesed.

The measurement system used by us is a new method, but relativelly similar to other conventional 2D records, made in models (19,20). We present a new plane traced trough the so called bone WALA points (modify of Andrews (8) soft Wala point) to avoid measurements problems produced by jaw asymmetries.

## Conclusions

The upper maxillary curve of Wilson at first molars in patients with bilateral posterior crossbite is more concave compared with normocclusive non orthodontic group or malocclusive without upper compression. It suggests no dentoalveolar compensations in bilateral crossbite patients.

The three groups showed no statistical differences concerning buccolingual inclinations of the second upper molar.

We are increasing the sample to find new criteria to establish the appropiate buccolingual inclination of the posterior teeth for treating orthodontic patients.

## References

[1] Motegi E, Miyazaki H, Ogura I, Konishi H, Sebata M (1992). An orthodontic study of temporomandibular joint disorders. Part 1: epidemiological research in Japanese 6-18 years old. Angle Orthod.

[2] Thilander B, Rubio G, Pena L, De Mayorga C (2002). Prevalence of Temporomandibular Dysfunction and Its Association With Malocclusion in Children and Adolescents: An Epidemiologic Study Related to Specified Stages of Dental Development. Angle Orthod.

[3] Mohlin BO, Derweduwen K, Pilley R, Kingdon A, Shaw WC, Kenealy P (2004). Malocclusion and Temporomandibular Disorder: A Comparison of Adolescents with Moderate to Severe Dysfunction with those without Signs and Symptoms of Temporomandibular Disorder and Their Further Development to 30 Years of Age. Angle Orthod.

[4] John MT, Hirsch C, Drangsholt MT, Mancl CA y Setz1 JM (2002). Overbite and Overjet are not Related to Self-report of Temporomandibular Disorder Symptoms. J Dent Re. s.

[5] Egermark I, Magnusson T, Carlsson G (2003). A 20-Year Follow-up of Signs and Symptoms of Temporomandibular Disorders and Malocclusions in Subjects With and Without Orthodontic. Treatment in Childhood. Angle Orthod.

[6] Schuyler C (1947). Correction of occlusion; disharmony of the natural dentition. N Y State Dent J.

[7] Beyron H (1969). Optimal occlusion. Dent Clin North Am.

[8] Andrews F (1972). L. The six Keys to normal Occlusion. Am J Orthod.

[9] Marshall S, Dawson D, Southard KA, Lee AN, Casko JS, Southard TE (2003). Transverse molar movements during growth. Am J Orthod Dentofacial Orthop.

[10] Handelman CS, Wang L, BeGole EA, Haas AJ Haas (2000). Non surgical Rapid Maxillary Expansion in Adults Reporto on 47 Cases using the Haas Expander. Angle Orthod.

[11] Ito H, Okimoto K, Mizumori T, Terada Y, Maruyama T (1997). A clinical study of the Relationship Between Occlusal Curvature and Craniomandibular Disorders. Int J Prosthodont.

[12] Alqerban A, Jacobs R, Lambrechts P, Loozen G, Willems G (2009). Root resorption of the maxillary lateral incisors caused by impacted canine: a literatura review. Clin Oral Investig.

[13] Wadhwa L, Utreja A, Tewari A (1993). A study of ical signs and synptoms of temporomandibular dysfunctionn in subjects with normal occlusion, untreated and treated malocclusions. Am J Orthod-Dentofacial-Orthop.

[14] Kohakura S, Kasia K, Ohno I, Kanazawa E (1997). Relationship between maxillofacial morphology and morphological characteristics of vertical sections of the mandible obtained by CT scanning. J Nihon Univ Sch Dent.

[15] Tsunori M, Mashita M, Kasia K (1998). Relationship between facial types and tooth and bone characteristics of the mandible obtained by CT scanning. Angle Orthod.

[16] Ross VA, Isaacson RJ, Germane N, Rubenstein LK (1990). Influence of vertical growth pattern on faciolingual inclinations and treatment mechanics. Am J Orthod Dentofacial Orthop.

[17] Ferrario VF, Sforza C, Poggio CE, Serrao G, Colombo A (1999). Three-dimensional dental arch curvature in human adolescents and adults. Am J Orthod Dentofacial Orthop.

[18] Ferrario VF, Sforza C, Colombo A, Ciusa V, Serrao G (2001). Threedimensional inclination of the dental axes in healthy permanent dentitionsâa cross sectional study in a normal population. Angle Orthod.

[19] Weissheimer A, de Menezes LM, Mezomo M, Dias DM, de Lima EM, Rizzatto SM (2011). Immediate effects of rapid maxillary expansion with Haas-type and hyrax-type expanders: A randomized clinical trial. Am J Orthod Dentofacial Orthop.

[20] Corbridge JK, Campbell PM, Taylor R, Ceen RF, Buschange PH (2011). Transverse dentoalveolar changes after slow maxillary expansiÃn. Am J Orthod Dentofacial Orthop.

